# Can number and size of offspring increase simultaneously? - a central life-history trade-off reconsidered

**DOI:** 10.1186/1471-2148-12-44

**Published:** 2012-03-31

**Authors:** Eero Schroderus, Minna Koivula, Esa Koskela, Tapio Mappes, Tuula A Oksanen, Tanja Poikonen

**Affiliations:** 1 Centre of Excellence in Evolutionary Research, Department of Biological and Environmental Science, University of Jyväskylä, P.O. Box 35, FI-40014 Jyväskylä, Finland; 2 MTT, Biotechnology and Food Research, Biometrical Genetics, FI-31600 Jokioinen, Finland; 3 Department of Biological and Environmental Science, University of Jyväskylä, P.O. Box 35, FI-40014 Jyväskylä, Finland

**Keywords:** *Myodes glareolus*, Litter size, Birth size, Genetic correlation, Heritability

## Abstract

**Background:**

To maximize their fitness, parents are assumed to allocate their resources optimally between number and size of offspring. Although this fundamental life-history trade-off has been subject to long standing interest, its genetic basis, especially in wild mammals, still remains unresolved. One important reason for this problem is that a large multigenerational pedigree is required to conduct a reliable analysis of this trade-off.

**Results:**

We used the REML-animal model to estimate genetic parameters for litter size and individual birth size for a common Palearctic small mammal, the bank vole (*Myodes glareolus*). Even though a phenotypic trade-off between offspring number and size was evident, it was not explained by a genetic trade-off, but rather by negative correlations in permanent and temporary environmental effects. In fact, even positive genetic correlations were estimated between direct genetic effects for offspring number and size indicating that genetic variation in these two traits is not necessarily antagonistic in mammals.

**Conclusions:**

Our results have notable implications for the study of the life-history trade-off between offspring number and size in mammals. The estimated genetic correlations suggest that evolution of offspring number and size in polytocous mammals is not constrained by the trade-off caused by antagonistic selection responses *per se*, but rather by the opposing correlative selection responses in direct and maternal genetic effects for birth size.

## Background

Fitness is determined by the number of offspring that reproduce successfully. The probability of offspring to reproduce in time can be increased with a larger investment per offspring, which inevitably decreases offspring number [1]. This fundamental life-history trade-off between offspring number and quality (which is most commonly measured as body size) is derived from the allocation of limited parental resources during a single reproductive attempt, such as energy and abdominal space [2]. Moreover, offspring size can be constrained by pelvic size and shape [3,4]. It is crucial to recognize whether this phenotypic trade-off between offspring number and size is due to a negative genetic correlation, since that would constrain the short term evolution of these central life-history traits. In theory, genetic correlations between life-history traits are expected to be negative [5], however, they have frequently been estimated as positive [6].

A negative genetic correlation between offspring number and size has been reported in oviparous vertebrates (fish [7,8], reptiles [9,10] and birds [11]). In contrast to these taxa in which offspring (egg) size is purely a maternal character, in mammals, the determination of the offspring size is more complicated. Offspring birth size in mammals is influenced by both the phenotype of the mother (maternal effects) and genes of the offspring. Maternal effects for birth size cover numerous factors that influence nutrient supply to the foetus (such as uterine capacity and blood flow) and are expected to have a substantial effect on offspring birth size [12,13]. Maternal effects themselves can be heritable or environmentally induced; the latter can either influence a single reproductive event or span over several reproductive bouts [14]. Furthermore, maternal genetic effects can increase or decrease the potential of populations to respond to selection on offspring size depending on the correlation between direct and maternal genetic effects [15,16]. A large direct genetic effect (genes in the offspring) on birth size would decrease the maternal flexibility in resource allocation between the number and size of the offspring in mammals. Thus, when estimating genetic parameters it is important to use individual records for birth size traits, together with the modelling of direct and maternal genetic effects [17]. However, this causes the trade-off at the genetic level to be divided into correlations between offspring number and two separate genetic effects (direct and maternal) for birth size, which makes the estimation and interpretation of the results challenging. Given the complexity of this offspring number-offspring size trade-off in mammals, it is not surprising that only a few studies have attempted to determine the genetic basis of the number-size trade-off in mammals [14,18,19].

As a litter bearing small mammal, the bank vole (*Myodes glareolus*) is ideally suited for the study of the offspring size-number trade-off. Bank voles can be bred intensively in the laboratory which allows for the collection of a deep and large pedigree, which is necessary to efficiently study maternal genetic effects [20]. A common garden experiment also avoids complications in the estimation of the genetic parameters created by environmental heterogeneity [21,22]. The use of the animal model ensures that estimated genetic parameters refer to a wild caught base population. The animal model is a flexible method to estimate variance components due to different sources without the need for complicated breeding designs. It utilizes all the information from the pedigree, takes selection into account and, under the infinitesimal model, gives unbiased estimates of the base population [20].

Here we report, to the best of our knowledge, the first estimation of the genetic correlation between offspring number and individual offspring size in a polytocous wild species. Our analysis is based on a large (over 10 000 animals) pedigreed laboratory colony founded by wild-caught bank voles. It shows how direct, maternal genetic and environmental effects contribute to the phenotypic trade-off between offspring number and size at birth in small mammals.

## Methods

### Study species and data recording

The bank vole is a common mammalian species in the Palearctic region [23]. In central Finland, females produce up to four litters of 1-9 pups during the breeding season, and there is substantial variation in both litter size and offspring size both among females and between litters of the same female [24]. Both the number and the size of offspring at birth are important fitness components. The size of offspring indicates quality, as it correlates positively with survival and breeding success [19,25,26], while litter size is adjusted to environmental conditions and is subject to balancing selection [25,27,28].

A laboratory population was established from wild individuals captured in Konnevesi, central Finland, during the summer of 2000 and subjected to artificial selection toward small and large litter sizes. Selection lines were founded from 150 females and 116 males. Both lines were pooled together in the analysis. All founder males were wild-caught, while some of the "founder" females were laboratory-born offspring of wild-trapped individuals (and thus had known parents). The selection procedure was a combination of between- and within-family selections. Litter size records were collected from generations 1-5 and birth size records were taken from generations 2-6. 1025, 874, 863 and 83 females had 1, 2, 3 and 4 litters respectively.

Animals were housed in standard mouse cages and maintained in a 16 L:8D photoperiod at 20 ± 2°C. Wood shavings and hay were provided as bedding, while food (labfor 36, Lactamin AB, Stockholm, Sweden) and water were available *ad libitum*. Pregnant females were checked once a day for parturition. After parturition, the birth size was measured using an electronic scale (± 0.01 g) and head width with a stereomicroscope.

The use of the animals adhered to ethical guidelines for animal research in Finland (The Finnish Act on Animal Experimentation, 62/2006) as well as the institutional guidelines. The study was conducted under permissions from the National Animal Experiment Board.

### Statistical analysis

Statistical significance of fixed factors was initially studied with univariate models using SPSS statistical software (version 18.0). All random effects were excluded except for the residual and 'individual' for the litter size. Fixed effects fitted for the birth mass were the number of parity (four classes) and sex (two classes); for head width, the factors were sex and measurer (ten classes); for litter size, the factors were parity, age (in days) nested as a linear covariate within the parity. Alternatively in the univariate analysis, the size of the birth litter was used as a covariate for birth mass and head width to estimate variance components after the effect of litter size was removed. The effect of inbreeding on the studied traits was found to be statistically non-significant.

The (co)variance components were estimated with the average information Restricted Maximum Likelihood (REML) -procedure using ASReml version 2.0 [29,30].

The following linear models were used:

Birth mass and head width:

$$y_{1}=X_{1}b_{1}+Z_{1}a_{1}+\text{Mm}+\text{Nn}+\text{Lc}+e_{1}$$

Litter size:

$$y_{2}=X_{2}b_{2}+Z_{2}a_{2}+\text{Qq}+e_{2}$$

In which **y_1 _**and **y_2 _**are the vectors of phenotypic observations for birth mass/head width and litter size; **b_1 _**and **b_2 _**are the vectors of fixed effects for birth size and litter size; **a_1_**, **a_2 _**and **m **are the vectors of direct additive genetic effects for birth size, litter size and maternal additive genetic effects for birth size; **n **and **q **are the vectors of maternal permanent effects for birth size (non-genetically determined effects that the mother has on all her offspring in all litters) and permanent individual effects for litter size (non-genetic effect on sizes of all litters of one female); **c **and **k **are the vectors of litter effects for birth size (environmental effect common for all the offspring in one litter) and temporary environmental effect for litter size (described later); finally, **e_1 _**and **e_2 _**are the vectors of residuals for birth size and litter size respectively. Fixed and random effects are fitted to individual records by incidence matrices **X_1_**, ***X*_2_**, **Z_1, _Z_2, _M, N, Q**, **L **and **K**.

$\text{E}\left[\begin{array}{c} y_{1}\\ y_{2} \end{array}\right]=\left[\begin{array}{cc} X_{1} & b_{1}\\ X_{2} & b_{2} \end{array}\right]$ and the expectations of random effects are zero.

Variances and covariances:

$$\text{Var}\;\left[\begin{array}{c} a_{1}\\ a_{2}\\ m\\ n\\ q\\ c\\ k\\ e_{1}\\ e_{2} \end{array}\right]=\left[\begin{array}{ccccccccc} A\sigma_{a1}^{2} & A\sigma_{a1a2} & A\sigma_{a1m} & 0 & 0 & 0 & 0 & 0 & 0\\ A\sigma_{a1a2} & A\sigma_{a2}^{2} & A\sigma_{a2m} & 0 & 0 & 0 & 0 & 0 & 0\\ A\sigma_{a1m} & A\sigma_{a2m} & A\sigma_{m}^{2} & 0 & 0 & 0 & 0 & 0 & 0\\ 0 & 0 & 0 & I\sigma_{n}^{2} & I\sigma_{nq} & 0 & 0 & 0 & 0\\ 0 & 0 & 0 & I\sigma_{nq} & I\sigma_{q}^{2} & 0 & 0 & 0 & 0\\ 0 & 0 & 0 & 0 & 0 & I\sigma_{c}^{2} & I\sigma_{ck} & 0 & 0\\ 0 & 0 & 0 & 0 & 0 & I\sigma_{ck} & I\sigma_{k}^{2} & 0 & 0\\ 0 & 0 & 0 & 0 & 0 & 0 & 0 & I\sigma_{e1}^{2} & 0\\ 0 & 0 & 0 & 0 & 0 & 0 & 0 & 0 & I\sigma_{e2}^{2} \end{array}\right]$$

In which **A **and **I **are the numerator relationship matrix and identity matrix respectively. σ^2^_a1_, σ^2^_a2 _and σ^2^_m _are the direct additive genetic variance for birth size and litter size and maternal additive genetic variance for birth size; σ^2^_n _and σ^2^_q _are the permanent environmental maternal variance for birth size and permanent individual variance for litter size; σ^2^_c _and σ^2^_k _are the common litter variance for birth size and temporary environmental variance for litter size; σ^2^_e1 _and σ^2^_e2 _are the residual variances for the birth size trait and litter size. σ_a1a2_, σ_a1m _and σ_a2m _are the additive genetic covariances between corresponding genetic effects; σ_nq _is the covariance between permanent maternal effects for birth size and permanent individual effects for litter size; σ_ck _is the covariance between common litter effects for birth size and the temporary environmental effect for litter size. In the univariate models, direct-maternal genetic covariance for birth size traits was set to zero because models including covariance did not converge. In the bivariate analysis, the residual covariance was set to zero because the dataset was composed of two separate parts: one containing records for litter size and the other for birth mass and head width. However, due to the functional relationship between litter size and offspring size, residual variation in the former is presumably correlated with the common litter environment of the latter. For that reason, actual residual variance of litter size was fixed to 0.01, and a dummy 'temporary environmental effect' was fitted for litter size in the bivariate models which was then allowed to correlate with the fitted litter effect for offspring. Without this temporary environmental effect for litter size, the correlation between permanent environmental effects tended to converge outside the parameter space.

Estimates of the ratios of variance components to the total phenotypic variance were calculated as: (heritability) h^2 ^= Va/Vp; (maternal heritability) m^2 ^= Vm/Vp; (maternal permanent environmental effect) n^2 ^= Vn/Vp; (common litter effect) c^2 ^= Vc/Vp; (permanent individual effect) q^2 ^= Vq/Vp, in which the total phenotypic variance (Vp) was determined as a sum of the appropriate (co)variance components.

Statistical significance of the genetic and environmental covariances was assessed with Log Likelihood ratio tests by comparing a full model with a model in which tested covariance was constrained to zero.

## Results

There was large variation in both offspring number and offspring size (body mass and head width) at birth. The coefficient of variation was largest in the litter size and lowest in the head width at birth (Table 1). The average litter size and offspring birth mass of the study population were close to values we have earlier observed in nature (5.3 pups, 1.76 g) [24]. Phenotypic correlations between litter size and mean offspring birth size were moderately negative (birth mass r = -0.46, *P *< 0.001; birth head width r = -0.35, *P *< 0.001) and similar to estimates from a natural population (-0.41 and -0.38 respectively) [19].

**Table 1:** Number of observations (n), trait means (± SD), coefficients of variation (CV) and range

Trait	n	Mean	CV	Range
Birth mass (g)	10986	1.89 ± 0.22	0.11	0.84-3.28

Head width (mm)	10971	8.22 ± 0.38	0.05	5.78-10.56

Litter size	2665	4.48 ± 1.53	0.34	1-9

Estimates of direct heritability (h^2^) from the univariate models were low: 0.10 ± 0.03 for litter size, 0.08 ± 0.03 for birth mass and 0.07 ± 0.03 for birth head width (Tables 2, 3 and 4 respectively). The permanent individual effect (q^2^) for litter size was 0.14 ± 0.03. The litter effect (c^2^) explained approximately half of the phenotypic variance in the birth size. Maternal heritabilities (m^2^) were 0.09 ± 0.03 for birth mass and 0.03 ± 0.02 for birth head width. Permanent maternal effects (n^2^) were 0.08 ± 0.03 for birth mass and 0.06 ± 0.02 for birth head width, while litter effects were 0.45 ± 0.02 and 0.49 ± 0.02, respectively. Adjusting the birth mass and head width for size of the birth litter decreased total phenotypic variance by 19% in the birth mass and 13% in the birth head width. The variance due to a common litter and maternal permanent environment decreased with the adjustment, whereas direct genetic variance increased. When birth mass and head width were analyzed in bivariate models with litter size, there were only minor differences in variance components compared to those derived from univariate models.

**Table 2:** Estimated variance components, heritabilities (h^2^) and permanent animal effects (q^2^) for litter size (Standard error)

	Univariate	Bivariate	
		**With birth mass**	**With birth head width**

Vp	2.011 (0.059)	2.013 (0.059)	2.011 (0.059)

Va	0.193 (0.055)	0.194 (0.054)	0.194 (0.054)

Vq	0.279 (0.062)	0.274 (0.061)	0.270 (0.061)

h^2^	0.10 (0.03)	0.10 (0.03)	0.10 (0.03)

q^2^	0.14 (0.03)	0.14 (0.03)	0.13 (0.03)

Vp = phenotypic variance; Va = additive genetic variance; Vq = permanent individual effect variance

**Table 3:** Estimated variance components, heritabilities (h^2^), common litter effects (c^2^), maternal heritabilities (m^2^) and permanent maternal effects (n^2^) for birth mass (Standard error)

	Univariate		Bivariate
	**Not adjusted for litter size**	**Adjusted for** **litter size**	**(with litter size)**

Vp	0.054 (0.001)	0.044 (0.001)	0.055 (0.001)

Va	0.004 (0.002)	0.005 (0.002)	0.005 (0.002)

Vm	0.005 (0.002)	0.005 (0.001)	0.004 (0.002)

Vc	0.024 (0.001)	0.014 (0.001)	0.025 (0.001)

Vn	0.004 (0.001)	0.004 (0.001)	0.004 (0.001)

h^2^	0.08 (0.03)	0.11 (0.03)	0.09 (0.03)

c^2^	0.45 (0.02)	0.32 (0.02)	0.44 (0.02)

m^2^	0.09 (0.03)	0.11 (0.03)	0.08 (0.03)

n^2^	0.08 (0.03)	0.08 (0.03)	0.08 (0.03)

Vp = phenotypic variance; Va = additive genetic variance; Vc = common litter variance; Vm = maternal genetic variance; Vn = maternal permanent environmental variance

Direct genetic correlations between litter size and birth size traits were positive (birth mass 0.54 ± 0.23; birth head width 0.47 ± 0.26), whereas the correlations between direct genetic effects for litter size and maternal genetic effects for birth size were negative (birth mass -0.30 ± 0.23; birth head width -0.47 ± 0.38) (Table 5). The latter were statistically non-significant. Correlations between direct and maternal genetic effects in both the birth mass and head width were weakly positive (0.04 ± 0.32 and 0.34 ± 0.76) but statistically non-significant.

In both birth size traits, the temporary environmental correlations between litter size and birth size traits were significantly negative (Table 5). The correlation between permanent individual and maternal effects was -0.35 ± 0.17 between litter size and birth mass and -0.44 ± 0.17 between litter size and birth head width. The correlations between the temporary environmental effect for litter size and litter effects for birth size were -0.67 ± 0.02 and -0.52 ± 0.02 respectively.

**Table 4:** Estimated variance components, heritabilities (h^2^), common litter effects (c^2^), maternal heritabilities (m^2^) and permanent maternal effects (n^2^) for head width at birth (Standard error).

	Univariate		Bivariate
	**Not adjusted for litter size**	**Adjusted for litter size**	**(with litter size)**

Vp	0.134 (0.003)	0.117 (0.003)	0.137 (0.003)

Va	0.010 (0.004)	0.013 (0.004)	0.010 (0.005)

Vm	0.005 (0.003)	0.005 (0.003)	0.003 (0.004)

Vc	0.066 (0.003)	0.050 (0.002)	0.068 (0.003)

Vn	0.008 (0.003)	0.005 (0.003)	0.009 (0.003)

h^2^	0.07 (0.03)	0.11 (0.03)	0.07 (0.03)

c^2^	0.49 (0.02)	0.43 (0.02)	0.49 (0.02)

m^2^	0.03 (0.02)	0.04 (0.02)	0.02 (0.03)

n^2^	0.06 (0.02)	0.04 (0.02)	0.06 (0.02)

Vp = phenotypic variance; Va = additive genetic variance; Vc = common litter variance; Vm = maternal genetic variance; Vn = maternal permanent environmental variance

**Table 5:** Genetic and environmental correlations between litter size and birth size traits (standard error)

	Birth mass	*χ*^2^	Birth head width	*χ*^2^
Genetic correlations				

LS direct - BS direct	0.54 (0.23)*	5.00	0.47 (0.26)*	3.34

LS direct - BS maternal	-0.30 (0.23)	1.20	-0.47 (0.38)	0.30

BS direct - BS maternal	0.04 (0.32)	0.00	0.34 (0.76)	1.26

Environmental correlations				

Permanent environmental	-0.35 (0.17)*	3.00	-0.44 (0.17)*	4.32

LS residual - BS litter	-0.67 (0.02)***	635.40	-0.52 (0.02)***	345.22

LS litter size; BS birth size (mass or head width)

Statistical significance: * *P *< 0.1; **P < 0.01;***P < 0.001

*χ*^2 ^Chi square test statistic for loglikelihood-ratio test of the covariance

## Discussion

In this study, we quantified the genetic basis of a fundamental life-history trade-off between offspring number and size. This is essential, as understanding the relative influence of genetic versus environmental causes behind this important phenotypic trade-off is necessary for predicting the strength and direction of evolution. By using a sufficiently deep pedigree, we found that the phenotypic trade-off between offspring number and size in the bank vole was due to environmental effects rather than additive genetic effects. Our results emphasize that despite being functionally bound to a phenotypic trade-off, the common genetic basis of litter size and birth size is comprised of antagonistic as well as parallel genetic variation.

### Heritability of litter size

The heritability of litter size was low, but similar to estimates of *h^2 ^*in other polytocous mammals [31-35]. This is expected since litter size is a composite trait, with ovulation rate setting the maximum value, while the number of offspring is further influenced by fertilization, implantation and embryonic mortality. Irrespective of the underlying genetic component of litter size, these effects add environmental variation to the total phenotypic variation in litter size. Indeed, the heritability of the ovulation rate is greater than the heritability of litter size in several vertebrates (swine [36] and mice [37]). Previous estimates of litter size heritability in bank voles were substantially overestimated [19], most likely because of the smaller sample size and methods used (i.e. mother-daughter regression). Heritability estimates based on mother-daughter regression can be four times larger than animal model estimates of h^2 ^[33].

### Sources of variation in offspring size at birth

Direct genetic effects that describe the genetic potential for a foetus to grow and absorb nutrients through the placenta explained 7-8% of the phenotypic variation in both birth size traits. These values are comparable to estimates of h^2 ^reported for birth size in other mammals [14,17,38]. Birth size is expected to be largely determined by maternal effects, and, including a litter effect, the overall combination of maternal effects accounted for 58-62% of phenotypic variation. However, only a small proportion of maternal effects was explained by additive genetic effects, since the maternal heritability was only 9% in birth mass and 3% in birth head width. This is a bit surprising as selection is less efficient on maternal genetic effects compared to direct genetic effects [39]. Adjusting birth size for natal litter size reduced the variance explained by the litter effect and permanent maternal effect. This result was expected since the low heritability of litter size indicates that the variation in litter size was mainly due to environmental factors; removing the effect of litter size on birth size should therefore decrease the amount of environmental variation in birth size. Moreover, when adjusted for natal litter size, more variation was removed from birth mass than from birth head width. This demonstrates the more substantial trade-off between litter size and birth mass as was observed already from the phenotypic correlations.

### Genetic basis of resource allocation between offspring number and size

Our estimation of co-variation between different genetic and environmental effects showed only weak support for a genetic trade-off between litter size and offspring size. The genetic correlation between litter size and direct genetic effects for birth size was positive, which indicates that the genes that increase female litter size tend to also enhance that individual's size at birth. Previous reports of a negative genetic correlation between litter size and mean offspring birth size in a bank vole [19] do not disagree with the present results (see Table 5). The previous study estimated the correlation only between the litter size and maternal genetic effects for birth size (here proven to be negative) and ignored the positive correlation between litter size and direct genetic effects for birth size.

Other studies have reported both negative [14] and positive [38] estimates for the maternal environmental correlation between litter size and birth size. A positive environmental correlation could arise if the environment affects the traits through resource acquisition [40]. For example, in the case of offspring number and size, abundant nutrition that causes ovulation of extra eggs allows mothers to support the growth of large foetuses. Conversely, a negative environmental correlation is expected if the environmental source of variation in litter size does not affect total maternal reproductive resources. The latter case is likely to happen in the bank vole, in which permanent and temporary environmental correlations between litter size and offspring size were strongly negative. The bank vole has an extremely variable litter size [19,24], and large environmental variation in the litter size demonstrated by a low heritability (this study). As a small mammal, the bank vole is an income breeder whose capacity to support growth of the foetuses during late pregnancy is not likely to be connected with environmental variation affecting offspring number, which is already determined early in pregnancy.

### Selection for offspring number and size

In general, selection on offspring size at birth and litter size acts antagonistically on the mother and offspring [19,25,41-43]. This combined with a presumed negative genetic correlation between offspring number and size is thought to constrain the evolution of these traits [44]. Our results indicate that genetic variation in offspring viability and offspring number are not necessarily antagonistic in mammals. A positive genetic correlation between direct genetic effects for litter size and offspring birth size can reduce parent-offspring conflict in offspring size as the same genes increase fitness at both levels. Also, an effectively null correlation between direct and maternal genetic effects for birth size implies a low level of parent-offspring conflict in the bank vole. Genetic correlations in natural populations of bank voles should be stronger than those estimated here, since genetic correlations are typically weaker in good environments such as laboratory conditions [22,45].

These data were obtained using a population that has been subject to short-term, two-way selection for litter size. Under an infinitesimal model and with complete pedigree information, the animal model takes selection into account when the selective events are included in the data set [20]. The infinitesimal model, whereby quantitative genetic variation is explained with a large number of unlinked genes of small effect, is not a realistic assumption but it does work reasonably well for short-term selection experiments [46]. In our selection experiment, litter size in the two lines (towards small and large) has diverged. However, divergence in offspring size has not been so straightforward, thus demonstrating the complex nature of this important life-history trade-off. (Schroderus, Koivula, Koskela, Mappes, Oksanen and Poikonen; Unpublished data).

## Conclusions

We have shown that the phenotypic trade-off observed between offspring number and size in a polytocous small mammal was due to environmental effects rather than additive genetic effects. Our results indicate that genetic variation in offspring number and size are not necessarily antagonistic in mammals. This finding is in line with the many positive estimates of genetic correlations reported between life-history traits [6]. Our results emphasize the complex nature of offspring number - size trade-off in mammals in terms of both environmental and genetic variation. The structure of the additive genetic covariance matrix suggests that evolution of offspring number and size in polytocous mammals may not be constrained by the trade-off *per se *caused by antagonistic selection responses, but rather by the opposing correlative selection responses in direct and maternal genetic effects for birth size.

## Competing interests

The authors declare that they have no competing interests.

## Authors' contributions

ES participated in the collection of the data, performed statistical analysis and wrote the manuscript. All other authors participated in the collection of the data, and contributed and approved the final manuscript.
